# Midwives in Health Sciences as a Sociocultural Phenomenon: Legislation, Training and Health (XV–XVIII Centuries)

**DOI:** 10.3390/medicina58091309

**Published:** 2022-09-19

**Authors:** Blanca Espina-Jerez, Laura Romera-Álvarez, Maylene Cotto-Andino, Mercedes de Dios Aguado, José Siles-Gonzalez, Sagrario Gómez-Cantarino

**Affiliations:** 1 Department of Nursing, University of Alicante, Carretera de San Vicente del Raspeig s/n, 03690 Alicante, Spain; 2 ENDOCU Research Group (Nursing, Pain and Care), University of Castilla-La Mancha, 45071 Toledo, Spain; 3 Faculty of Physiotherapy and Nursing, Toledo Campus, University of Castilla-La Mancha, 45071 Toledo, Spain; 4 Centre of Language, University of Castilla-La Mancha, 45071 Toledo, Spain; 5 Health Sciences Research Unit: Nursing (UICISA: E), Nursing School of Coimbra (ESEnfC), Avenida Bissaya Barreto s/n, 3004-011 Coimbra, Portugal

**Keywords:** history of nursing, history of medicine, midwifery, witchcraft, legislation and jurisprudence, gender and health, sociology of health and illness, education in health humanities

## Abstract

*Background and Objectives:* The first inquisitorial processes were developed against Muslims and Jews. Then, they focused on women, especially those dedicated to care. Progressively, they were linked to witchcraft and sorcery due to their great assistance, generational and empirical knowledge. The health historiography of the 15th–18th centuries still has important bibliographic and interpretive gaps in the care provided by women. The main objective was to analyse the care provided by midwives in the legislative and socio-sanitary context of New Castile, in the inquisitorial Spain of the 15th–18th centuries. *Materials and Methods*: A historical review was conducted, following the Dialectical Structural Model of Care. Historical manuals, articles and databases were analysed. *Results*: The Catholic Monarchs established health profession regulations in 1477, including midwives. However, all legislations were annulled by Felipe II in 1576. These were not resumed until 1750. Midwives assumed a huge range of functions in their care commitment (teaching, care and religion) and were valued in opposing ways. However, many of them were persecuted and condemned by the Inquisition. They used to accompany therapeutic action with prayers and charms. Midwives were usually women in a social vulnerability situation, who did not comply with social stereotypes. *Conclusions*: Midwives, forerunners of current nursing and health sciences, overcame sociocultural difficulties, although they were condemned for it. Midwives achieved an accredited title, which was taken from them for two centuries. They acted as health agents in a society that demanded them while participating in a “witch hunt”.

## 1. Introduction

Ignorance, increasingly latent in the European Modern Age (15th–18th century), acted as a driving force behind the fear surrounding health, generating in popular discourses and linking the experience of ignorance and amazement in the face of healing. For the predecessors of al-Andalus or the Andalusians, science and religion did not represent a dogmatic antithesis to each other, but Muhammad himself encouraged the cultivation of medicine after religion [1]. Even the pre-15th century Christians of northern Spain themselves did not see it as a threat to the faith of individuals [2].

Thus, the Andalusians took up classical knowledge and made progress in the sciences of care and medicine during the almost eight centuries of their stay in the Iberian Peninsula (8th–15th century). After the Christian conquest, Arabs and Jews were linked to the sciences of health and care, which in turn linked the stigma of the cult to that which concerned their people, creating a need to “cleanse” the population. Thus, the science–religion binomial was joined by magic and witchcraft. This did not happen immediately but required a whole process of gestation. For example, the court of Alfonso X (13th century) was and is particularly known for its preservation of knowledge, which is why the Toledo translators’ project was promoted. However, it is curious how in these translations from Arabic into Spanish, the original names of the Andalusian authors and the translator himself were omitted, with the exception of notable scientists such as Avicenna, Averroes or Azarquiel [2].

With the Bull issued on 1 November 1478, Pope Sixtus IV authorised the Catholic Monarchs to proceed against heretics and fakers through the inquisitorial process. However, what influence did the Tribunal of the Holy Office have on social control and health sciences from that time until its end in 1820? Because of its politico-religious status, it was supposed to restore Catholic unity. However, once this objective was achieved, the Holy Office focused and expanded its persecution on a large number of women, many of whom were involved in health care.

At the end of the late Middle Ages and during the Modern Age (14th–18th centuries), the woman was structured in the collective imagination as a figure that inspired fear. The origin of this fear was none other than the very mystery of female nature, which posed a threat. The inquisitorial process against women linked to physical and psychological care, although existing in previous periods, became even more prevalent in the 17th and 18th centuries [3,4].

Both the personal and concrete experience, as well as the use and social construction of the image that was generated of women dedicated to health and care during the 14th–18th centuries, defined a historical condition that must be seen from three angles: (1) scientific, and the consequent rejection that women were part of this world; (2) religious, where the Christian tradition was an instrument of judgement and redemption; (3) legal, as an instrument of repression and punishment of dissident conduct [5].

The progressive organisation of power, strongly linked to the ecclesiastical world, resulted in a system that became increasingly intolerant and fearful, thus more rigorous and inflexible towards uncontrolled spaces and discordant positions within Christianity. Many women were accused and condemned for witchcraft and sorcery, precisely because of their great knowledge of healing plants, as well as for their assistance to the unknown collective of the time, the feminine.

Studies linking the Inquisition and health tend to focus on doctors, barbers and bleeders [6,7,8], few address women’s perspective on health and human care [9,10,11,12].

For this reason, the present research study acquires an important scientific relevance as it attempts to contribute to filling an existing gap in the history of nursing and health sciences, that of women dedicated to care in the Modern Age (15th–18th centuries). In order to do so, it is essential to resort to primary sources that can provide a version as close as possible to the history of midwives, witches and sorceresses, from what is closest to the truth, their testimonies.

The following section presents the methodology used to carry out the analysis in accordance with the research objectives. The main objective is to analyse the care provided by midwives in the legislative and socio-sanitary context of New Castile, in the inquisitorial Spain of the 15th–18th centuries. The secondary objectives are: (1) to learn about the regulations governing the care professions exercised by women in the Modern Age; (2) to describe the appearance of other figures in the field of female assistance; (3) to analyse the functions exercised by midwives, as well as the accusations made by the Inquisition for witchcraft, sorcery and superstition, in their care practice.

This is followed by a discussion in which the results are compared with previous and prior knowledge. Finally, the main conclusions are discussed.

## 2. Materials and Methods

### 2.1. Study Design

In this article, a review was carried out as a method to begin to address the subject of study. The aim was to analyse the care provided by midwives in the legislative and socio-sanitary context of New Castile, in the inquisitorial Spain of the 15th–18th centuries. Through the review, the scope of the existing body of literature on the specific topic was determined. It allowed the researchers to evaluate, synthesise and critique the evidence inherent to the objective of the study.

This was followed by recourse to various primary sources, specifically to documents preserved from one of the most important regions of the inquisitorial period, New Castile (Spain). Two main jurisdictions were grouped in this region: (1) the jurisdiction of the Holy Office of Toledo, which included the present-day dioceses of Toledo, Ciudad Real and Madrid-Alcalá; (2) the jurisdiction of the Holy Office of Cuenca, which brought together Cuenca and Sigüenza. All these districts were associated in what is known as New Castile.

The Dialectical Structural Model of Care (DSMC) was used. This model is suitable for studying the social and cultural history of care, which is fundamentally linked to the sexual and gender division of labour [13,14].

The DSMC makes it possible to analyse the structures of care in order to subsequently establish relationships among them. For this study, its application is of utmost importance, taking into account the social and cultural aspects that interact in the assistance and care provided by Castilian or New Castile midwives.

**Figure 1:**
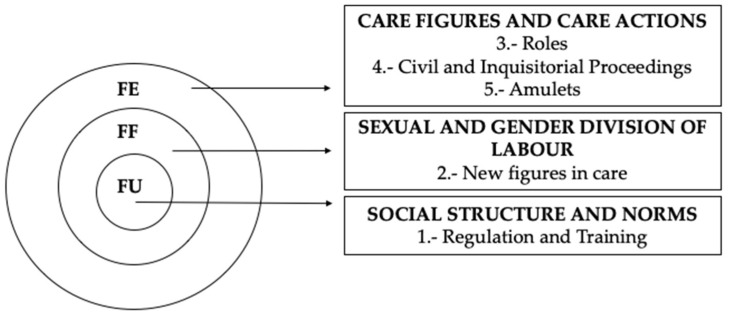
Theoretical DSMC model: application of its structures (FU, FF and FE) to data.

The structures used in the DSMC are: (1) Functional Unit (FU), which represents the social structure of coexistence and socialisation that transmits values, norms, beliefs, knowledge and feelings, through which social systems are constructed. In this case, it has to do with the regulation and training of the midwifery profession in the 15th–18th centuries and its repercussions at the socio-sanitary and professional levels. (2) Functional Framework (FF), which refers directly to the socio-sanitary context, which determines the sexual and gender division of labour, so decisive in the health and care professions. In this case, new figures of care appeared. (3) Functional Element (FE), integrates the social actors in charge of care, i.e., the health and the role of care figures, as well as the care actions themselves, which in this study are drawn from civil and inquisitorial processes [15]. This research proposes five thematic blocks analysed through the DSMC, each representing structures specific to the cultural and social history model (Figure 1) [16].

Source: The authors’ own elaboration through the application of the Dialectical Structural Model of Care (DSMC) [16].

### 2.2. Search Strategy

The review process began with an exploratory research question [17] aimed at systematically synthesising and critiquing existing knowledge [18], in this case: “What impact did the development of midwifery training in the 15th–18th centuries have on the possibilities and limits of midwifery care?

To answer this question, the DSMC was applied. The researchers agreed on the eligibility criteria, which had to contain information on the midwife in the Modern Age. The review included the regulation of the midwifery profession in relation to its training, taking into account the background of the period under study. In addition, the legislation for other emerging professional figures in the profession was reviewed; the functions of the midwife were analysed; and various inquisitorial processes that highlighted the care situation of these figures, as well as other experiences and methods accompanying the cure, were analysed.

The review included inquisitorial processes from the period under study, 15th–18th centuries, as well as peer-reviewed articles, official dissertations, proceedings and reports. It excluded conference proceedings, proposals and editorials. The documents consulted were in English, Spanish and French.

We began with an initial search aimed at finding out the background and delimitation of the subject of the study. In this phase, various databases were consulted: (1) PubMed; (2) Cochrane; (3) Bibliographic database on health in Latin America (CUIDEN); (4) Scopus; (5) Web of Science; (6) SciELO.

After this first search, we proceeded to consult manuals in physical and virtual format from different territories through the library service of the University of Castilla-La Mancha and Toledo Public Library. In order to have up-to-date information, the search was limited to the last 10 years. However, due to the fact that this is a historical topic and perhaps not so prone to bibliographical updating, earlier publications were consulted and selected because of their interest.

For the database exploration, a natural or free-text language, normalised and controlled with MeSH and DeCs descriptors, was used. These were combined with Boolean operators (“and/and”, “or/or”, “not/not”). The results obtained and used, as well as the search equations and filters used to arrive at them, are summarised in Table 1.

The inquisitorial cases and convictions of New Castile are essentially divided into two archives. The documents of the Courts of the Holy Office belonging to the archbishopric of Toledo are in the National Historical Archive (AHN), and those of the archbishopric of Cuenca are in the Diocesan Archive of Cuenca (ADC). For the consultation of documents held by the AHN, combined searches were carried out through the Portal of Spanish Archives (PARES), entering the following search terms and filtering by archive (AHN): “Institutions of the monarchy” and “Tribunal of the Inquisition of Toledo”. On the other hand, the search in the ADC was carried out manually using the Catalogue published by Dimas Pérez [4].

The terms used in the PARES search after filtering the archive (AHN), “Institutions of the monarchy” and “Court of the Inquisition of Toledo”, were: “midwife”, “delivery”, “midwife”, “midwife”, “healing”, “cures”, “sorcery”, “witchcraft”. It was not necessary to filter the period of study as the “Court of the Inquisition of Toledo” parameter itself already narrowed the search by date. Due to the few accusations found under the search term “midwife”, a broad investigation of this term was carried out, i.e., without filters, which resulted in the executions or civil lawsuits discussed later, as well as the case of the certification of the “child who had been born naturally circumcised”.

The search in the ADC followed a different procedure, as the catalogue is not digitised. A manual search was carried out, following the two existing records on the documents collected in the ADC, that of Sebastián Cirac Estopañán (1965) and that of Dimas Pérez Ramírez (1982), both contained in the same manual [4]. The chapters “Crime Processes” and “Fourth Series: Crime Processes, II” were reviewed. In the search, the same terms were used as in the AHN.

### 2.3. Review Process

The database search was carried out between October 2021 and January 2022. Archival research, as well as the analysis and transcription of the records found, took place between February and May 2022. This type of review aims to explore the existing evidence on a particular topic and historical period, as well as to identify and address gaps in the existing evidence.

The inclusion criteria were as follows: (1) documents that directly address the topic of study; (2) period of study between the 15th and 18th centuries; (3) full-text material; (4) manuals lendable by libraries; (5) complete inquisitorial and civil cases; (6) inquisitorial and civil cases limited to the jurisdictions of the archbishopric of Toledo and Cuenca; (7) women’s cases; (8) documents published in Spanish, English or French; (9) material published between 2012 and 2021.

The exclusion criteria applied were: (1) documents that considered the subject from different perspectives; (2) works included in earlier or later periods of study; (3) inquisitorial cases and convictions not pertaining to the archbishopric of Toledo and Cuenca, except for interesting exceptions; (4) convictions on grounds other than childbirth, or on charges other than sorcery, superstition or witchcraft; (5) material published before 2012, with the exception of works whose content was relevant to this study, as well as the primary sources themselves. Via consensus of the authors, articles, books, dictionaries, historical archival documentation and legislation were reviewed. A total of 57 documents were reached that met the inclusion and exclusion criteria.

### 2.4. Data Analysis

The documentary analysis was conducted from a qualitative perspective, systematically following the objective of the study. The steps followed in the analysis were: (1) thematic liaison; (2) preliminary classification of the documents based on inclusion and exclusion criteria; (3) selection of relevant information; (4) interpretation and comparison of the results. The selected material was analysed from the point of view of the five thematic study blocks, each of them encompassed within the DSMC structures: (1) training and regulation of the midwifery profession; (2) the emergence of new care figures; (3) functions of the midwife; (4) civil and inquisitorial proceedings against midwives; (5) amulets used in assisting pregnancy and birth. These blocks were contextualised in the Castilian population of the Modern Age (15th–18th centuries) in Castilla La Nueva (Spain). To extract and summarise the data, the first and second authors carried out a general data extraction. The third author examined the findings in depth. The fourth and fifth authors identified the thematic blocks encompassed in the DSMC structures, from the Functional Unit to the Functional Framework and the Functional Element. Discrepancies were resolved via consensus among the researchers. Thus, after working with all the material, it was possible to answer the initial question of this study: “what impact did the evolution of midwifery education in the 15th–18th centuries have on the possibilities and limits of midwifery care?”

## 3. Results

### 3.1. Formation and Regulation of the Midwifery Profession in the Modern Age (15th–18th)

#### 3.1.1. Background: Ordinances in the Late Middle Ages (13th–15th c.)

The first regulation of midwifery in Spain during this period dates from 1258, when the Court of Valladolid issued ordinances to prevent the mixing of races. Moorish and Jewish women were forbidden to care for Christian mothers and children, and vice versa, Christian women were forbidden to care for or raise Muslim or Jewish offspring [19].

In the same 13th century, Alfonso X the Wise [20] referred on many occasions to the office and the figure of the midwife in the *Siete Partidas*. In *Partida II*, he stated that the midwife should attend to the pregnant woman and the newborn, as well as the characteristics that the midwife, known as *obstetrix*, should have. In the *VI Partida*, he mentioned the “wise women”, those whose job was to assist in childbirth, the treatment of women’s own illnesses and some children’s ailments.

In this same period, *Cantigas de Santa María* (1252–1284) were published, also under the patronage of Alfonso X the Wise. They contain a number of miracles through the intercession of the Virgin, many of them linked to gynaecological issues such as abortion, caesarean section, sterility and infanticide [21].

Of particular interest is *Cantiga VII*, which narrates how a pregnant abbess is denounced by the nuns of her convent to a superior. After her devout prayer, the woman is helped by the Virgin Mary, who sends two angels to intercede for her. They performed a caesarean section in vivo, an unpermitted medical feat that was presented as a miracle. Thanks to the merciful intervention and power of the Virgin Mary, the abbess awoke and was able to exhibit her naked body before the bishop without the latter discovering her sin [22]. Although the caesarean section is depicted in vivo, it gains value from the manner in which the post-mortem intervention was performed: a right abdominal incision while in the lateral decubitus position [21,22]. In the Cortes of Zamora (1434) and in the Ordinances of Madrigal (1448), the free practice of midwives was approved [19,23].

#### 3.1.2. Regulation of the Care Professions Exercised by Women in the Modern Age (15th–18th c.)

Following the Madrigal Ordinances, on 30 March 1477, the Catholic Monarchs issued a Royal Pragmatic Decree creating the Royal Court of Protomedicine. With this, they also promoted the Health Regulations prior to their reign, in order to improve and organise the health professions. It functioned as a kind of official college of medicine and was made up of doctors and examining magistrates of the different professions, including physicists, surgeons, midwives, bleeders, barbers, apothecaries and embalmers. The Court also organised medical training, specifying the texts to be used. It examined and granted accreditation licences or diplomas and even delimited the competences of the above-mentioned health professions [23,24].

The Royal Court also visited apothecaries, supervised the preparation of medicines and decided on their authorisation or prohibition. It was responsible for the surveillance and study of epidemics and certain social problems and could impose sanctions. In general, it sought to cover medical care in the safest possible way in all its aspects, both in the Spain of the Catholic Monarchs and in the overseas dominions [23,24,25].

Years later, the Royal Protomedicine Ordinances of 1498 regulated the procedure for the legal practice of the health professions [19,23]. To do so, those who wished to work within the legal framework had to apply for a licence and pass a theoretical and practical examination. Thus, for example, in 1579, María de Herrera [26] requested in 1579 to be able to practice under the approval of her local protomedic:

*“Wife of Alonso de Zamora, a resident of the said town, and said that in order to be able to use the office of “comadre” (midwife) and algebraist and to cast “bizmas” (traditional medicines) and cure the bewildered, she needed to give information for the record to Doctor Olivares, his Majesty’s protomedic, resident in his Court, of how she uses the said office very well and faithfully and with good diligence and care and has done and does very good cures and cured and cures many poor people without taking any interest from them”* [26].

**Figure 2:**
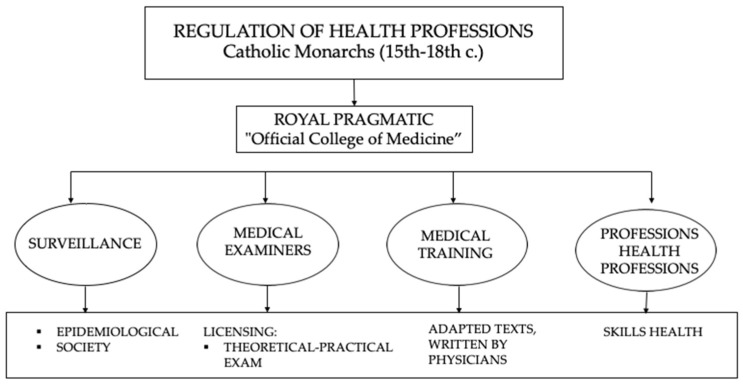
Regulation of the health professions: legislation, training and accreditation.

María de Herrera’s request shows that the examination was to be passed by midwives, healers and algebraists or experts in bone cures. Such was the case of María Hernández [27], who years earlier, in 1573, declared the following before the Royal Court of Protomedicine:

*“Wife of Lorencio de Vergara, neighbour of this town of Valladolid, I appear before your worship and say that, in order to present myself before his Majesty’s protomedic, so that he may give me licence and letter of examination to be able to cure in the office of algebraist, I need to make information and proof of the experience I have in the said art and of the cures that in this said town I have made and women, who through the favour and grace of Our Lord, have healed and remained healthy”* [27] (Figure 2).

Source: The authors’ own elaboration.

In María Hernández’s request for examination, there are also testimonies before the Royal Court that attested to the success of the applicant’s cures, for example, that of sawyer Juan de Grandas:

*“That there may have been five years, a little more or less time, that the said María Hernández cured this witness of a blow that a large beam gave him in the breasts while he was removing it from others, in such a way that this witness fell to the ground and the beam remained on top of him, from which the bones of his breasts were sunk and crippled, and the aforesaid woman cured him with plasters and soft things that she put on him, and other things, and she put his bones back in place, so that in 8–9 days that the aforesaid woman cured him, through the mercy and grace of Our Lord he was healthy and well”* [27].

Parallel to the Ordinances of 1498, which regulated the health professions, the prohibition of mixing Christians and Mudejars was dictated; therefore, women of both religions were forbidden to be midwives of the other group [28]. The new situation was published by Archbishop Hernando de Talavera in the following decree:

*“(...) that no Christian man or woman (...) should bathe in the baths of Moors or Moorish women, nor should Christian women give birth to Moorish midwives, although they may have Christian midwives (...)”* [29].

Years later, the exclusion of Moorish midwives was once again emphasised with the Council of Granada in 1565. These regulations had their origin in the fear of Moorish baptism, which consisted of circumcising newborns. This trait, considered by the Castilians to be characteristic of Islam, was also a feature of Judaism. However, Muslim midwives also performed other rites, such as the purification of the child, which they likewise tried to contain [30].

The fear was so widespread that in 1482, Vicente Almenara, a weaver from Valencia, went to the Inquisitor General of the province of Aragon to ask him to testify that his wife had given birth at home to a baby boy who had been born naturally circumcised. Vicente stated that he did this with the intention of preventing any future judaizing taint from falling on his son. The newborn was examined by a surgeon (Andrés Forcadell) and a midwife (Juana, wife of Francisco Coll, a barber). Both certified the anatomical normality of the baby’s genitals [31]. Once a body responsible for the organisation and functioning of the health professions had been created, and at the same time coinciding with the uniformity of Christian worship, the Catholic Monarchs, together with the Royal Court of Protomedicine, launched in 1541 the famous publication of the Book of the art of midwives or godmothers and of the regiment of pregnant women and children [32], which summarised the appropriate characteristics for midwives (Table 2).

### 3.2. Between Medicine and Health Sciences: Consolidation and the Emergence of a New Care Figure

However, the Modern Age left a two-century gap in Castile without regulated training and practice for some health professions. The examinations and corresponding licenses for midwives, spice merchants and druggists were abolished by Philip II in 1576 through a new Royal Pragmatic [33,34]. The King alluded that the protomedics conducted too many examinations in vain for spice merchants, midwives and embalmers, in addition to the many others who practised their trade outside the court not to be examined. Thus, he only allowed physicists, surgeons, apothecaries and barbers to be eligible for examination and professional licensing [35].

The new prohibition applied to the Kingdom of Castile and exempted those places governed by Particular Royalties, i.e., the Kingdom of Aragon and the city of Seville. In these territories, midwives continued to be examined and practised freely [35].

The absence of inspection for almost two centuries of the training, professional activity and organisation of competencies of midwives and woman healers left a significant trace of improvisation and malpractice in these trades. This situation led Joseph Suñol, president of the Court of Protomedicine, to expose to Ferdinand VI, through the Royal Council, the high rate of maternal and infant mortality and the serious public health problems that this entailed [36].

In view of the different consequences between the Kingdom of Castile and the Kingdom of Aragon and Seville, it was decided with the Royal Decree of 21 July 1750 to resume the supervision and aptitude examinations for the profession of midwife and midwife under the Instruction of the Protomedicate [19,36]. Thus, under the title *Exámen de parteros y parteras para poder ejercer su oficio, baxo la instruccion que estableciere el Protomedicato* [33], it was stated that midwives and midwives had to be examined according to the rules established by the Tribunal del Protomedicate, given the negative consequences of midwives with little skill and men who had taken up the profession of midwife without training (Table 3).

In this way, in addition to creating a regulation for the profession of midwife, it also hierarchically added that of surgeon-midwife, merging the classical art of midwifery with surgical and masculine knowledge. Prior to this time, medicine and surgery had been gaining ground in their consideration as a scientific and professional authority, which allowed them to attain important positions within the Protomedicate and to write treatises on obstetrics and small manuals [36].

Thus, along with the approval of the examination and degree licence came the publication of the “*Cartilla*”, *new, useful and necessary training for midwives, commonly known as “comadres” in the trade of* “*partear*” [35], with the aim of adequately training the midwives of the time. The treatise was written in vulgar language, as it was assumed that its readers were poorly literate. In order to take the examination, not only professional requirements had to be met (two years of practice) but also social (clean blood and baptismal certificate) and moral (good life and morals) requirements [36].

In 1780, the Royal College of Surgeons of Madrid was created, and in 1795, title twelve of the Ordinances of the Court of Protomedicine was dedicated to midwives, their examination and the duties of their practice [37]. However, the control of the Protomedicate by means of examinations and the issuing of diplomas was more important for the professional consolidation of surgery than for the professional improvement of the role and care of midwives [36]. An example of this was the case of Luisa Rosado in 1770–1771, an experienced midwife and worker at the School of the Forsaken of Atocha.

Luisa asked to exhibit a poster advertising her skills as a midwife. Even though she had obtained her matron’s degree, issued by the Protomedicate, and had years of experience, she received a refusal from the Royal Court to publish her poster. The report sent by the Protomedicate questioned her professionalism and warned that she could cause discomfort to surgeons and harm to women who put themselves in her hands. This midwife was advertising on her poster that she was capable of assisting certain complications in childbirth, a competence that had become the exclusive competence of the surgeon-midwife with the new regulations. Finally, Luisa managed to advertise her services after initiating a legal process with three petitions to the King [38,39]. This experience highlights the attitude of resistance of midwives of the time, such as Luisa Rosado, to the changes and loss of skills that the midwifery profession underwent.

### 3.3. Roles of the Midwife in the Modern Age: Teaching, Care and Religion

Obstetric–gynaecological care was not a field of interest for male figures dedicated to health until the middle of the 18th century [9].

The care of women’s health and illness was a practice of women for women that from the 16th–18th centuries was immersed in an institutional training vacuum but covered by a strong empirical, oral and transgenerational wisdom. As Harvey Graham [40] notes, childbirth assistance was a dirty job that only midwives could perform.

The apprenticeship of the trade was performed directly during the assistance of an expert midwife [41]. It is common to find this apprenticeship through family tradition or close relationships. The apprentices began at a young age and gradually replaced their teacher until they acquired sufficient training and skills to become independent [11,19].

The care function was the most developed of all. They assisted women during pregnancy, childbirth and puerperium, cared for the newborn, treated women’s own illnesses, advised on contraceptive measures and could even be called upon to perform abortions [19]. On occasions, the midwife would use utensils to facilitate childbirth or perform a caesarean section to ensure the baby’s survival or a post-mortem caesarean section [42]. However, the practice of caesarean section passed into the hands of the surgeon-midwife in 1750 [33], from which time the work of the midwife was consolidated, performing only autecological deliveries.

Another of the cases in which the midwife could perform a caesarean section was for the well-known emergency baptism [19,43]. So important was baptism after birth that another common practice in the late Middle Ages and early Modern Age was the rite of respite, the aim of which was to save the soul of the newborn. If the foetus was born lifeless, it was placed in front of the image of a saint or virgin, prayed to, and if the slightest sign of life appeared, real or fictitious, it was quickly baptised. This allowed him to be buried in a sacred place and prevented the accusation of having baptised a dead person [22,44].

However, the religious role of the midwife in the sacrament of baptism was not only limited to emergency situations. When the birth was successful, the parents granted the midwives the privilege of being the godmother of the baby and taking it to its christening; this was common among royal and noble circles [45]. This function is already reflected in the first manual of obstetrics written in Spanish, *Book of the art of the “comadres” or godmothers and regiment of the pregnant and giving birth and of the children* [32].

### 3.4. Civil and Inquisitorial Prosecutions of Midwives

Generally, accusations were related to childbirth assistance or were of a personal nature. In any case, the lawsuits found provided a wealth of information on the unstable situation and valuation of the profession (Table 4).

Midwives in their profession had access to the intimacy of women and had to keep professional secrecy. Haxa, a Muslim midwife, was denounced by Juana Ruiz for having publicly commented that after being widowed by her husband Alonso Ruiz de Medina, she had lived dishonestly and as a result had a daughter, whose birth Haxa attended. When the midwife was denounced, she was involved in a plot with Juana Ruiz for the goods that she received from her husband as a privilege for being the wife of noble sons [46,47].

Another curious case is that of María García, from Getafe (Madrid), who was accused of sorcery in the exercise of her profession; her accusation was followed as a civil lawsuit, and her sentence was mixed civil and inquisitorial. Her accuser accused her of spitting on the blood of a childbirth that had fallen on the ground. She was imprisoned and interrogated, she declared that she was a rustic and simple woman, that she was not superstitious by vice. It is possible that she sometimes believed, as it was said, that certain objects could cure certain illnesses, without her having malice or having committed a mortal sin, for which reason she should be acquitted and released.

However, the court condemned her. Her release from prison was to follow the usual protocol for a public act of faith: she was to go out naked from the waist up, mounted on a donkey with her hands and feet tied with a rope around her neck, while a crier exposed her crime in such a way as to serve both as punishment for the prisoner and as a source of fear for the spectators [48].

Another of the lawsuits involved an accusation of malpractice. Inés Martín, midwife and widow of Pedro Acevedo, was accused of having caused the death of Francisca Galiana and her child during childbirth. The complainants testified that Inés Martín had cut off the foetus’ arm, which was the cause of her death, and that when she saw this, the woman in labour also died. The midwife was imprisoned; the prosecution asked for the most severe penalty, and Inés asked for her freedom and acquittal for the abortion and death of Francisca.

She stated that Francisca had been exhausted from three days and three nights of labour, as the foetus was stuck in the woman’s pelvis. However, given her profession, she was supposed to help her give birth, because if she had not done so and left the mother to die, it could have cost her the same charge she eventually got. Because the foetus had already died during childbirth, she wanted to save the mother by removing the foetus, for which she had to cut off the foetus’ arm. After that, the mother must have died of haemorrhage.

Agnes claimed that she had more than 30 years of experience, during which she had been called upon by many women. On some occasions she had had to remove the babies with a “ladle” or “hook”, so that the mothers would not be in danger, which was lawful and authorised. She also confessed that she did not understand why Francisca’s relatives had denounced her, considering that during childbirth they had authorised such action. Despite her expertise in assisting complicated deliveries by means of instrumentation, Inés was convicted and sentenced [49].

In all three cases, it can be seen that they were all trained midwives. The first of them, Haxa, was involved in a personal plot with a conflict of interest over the widow’s pension. However, Haxa was acquitted. In the second case, Maria Garcia was accused of sorcery for the act of spitting on the ground coinciding with the blood of childbirth. Although she had no other accusations and she herself acknowledged that she was a simple, rustic farm woman with no superstitious intentions, she was harshly condemned by the judicial system. The third of them, Inés Martín, was accused of malpractice despite her proven experience and advanced obstetric training. She used instruments such as the forceps of the time, called a “hook”, or a “ladle”, which could well be what we know today as spatulas. The use of these instruments in complicated deliveries made it possible to save the lives of women and children, both in the times of Inés Martín (16th century) and from 1750 onwards, when this function was assumed solely and exclusively by the surgeon-midwife with far fewer prejudices and accusations.

### 3.5. Amulets in Pregnancy and Childbirth: The Human Experience of Health–Illness

The use of amulets and prayers to prevent various diseases and illnesses was widespread. In this sense, different aspects related to birth acquired special meanings. The placenta, the membranes and the umbilical cord were the object of superstition [50].

It was believed that the membranes forming the amniotic sac protected against drowning, favoured an easy delivery and brought good luck [50]. This protective function is corroborated by the long trial of faith opened in 1698 against Jaime Martí Tarazo, from the town of Castellón de la Plana (Castellón, Spain), who stole the “zurrón” (placenta) of a newborn baby. He claimed that the midwife had told him that the “zurrón” protected whoever had it with them from harm. Jaime was confident that it even protected “those who go to war” from “shotgun blasts”, a fact that he confirmed in his testimony with the story of the miller, José N., who also carried a “zurrón” and thus had already escaped shotgun blasts twice. The accused must have had such confidence in the placenta that one of the witnesses argued that Jaime gave a man a shotgun to shoot him to prove that he could not die. This is how this unknown man acted, firing up to six times, without any ammunition coming out, while when Jaime did, he did shoot himself. For the act of stealing a family’s bag, which other people also committed and for what was testified, Jaime Martí was accused of sorcery. However, the case does not provide any information on the final sentence and conviction in this process [51].

After the inventory of goods taken from the courtesan María de Acevedo, they found in her laboratory, among other things, a sample of the navel of one of her sons, which was probably part of his umbilical cord. María explained that she kept it because her “comadre” advised her to do so in order to cure the child’s eyes, in case they suffered from some ailment [52].

It was also a superstition among pregnant women that precious stones such as agate, sapphire and jasper had magical properties that would help the pregnancy to run smoothly and prevent pre-term births [53,54].

After birth, figs used to be hung on children because they were believed to have the virtue of warding off the evil eye. Similarly, they became part of women’s trousseau [54]. They were widely used by different social groups, decorating ordinary houses, but also those of nobles, bankers, confectioners and other tradesmen. They were made of a wide variety of materials, including precious stones [10].

## 4. Discussion

This research study analysed various primary and secondary sources on the legislation of the period under study, from the 15th to the 18th centuries, and its antecedents. It also explored the socio-sanitary framework in which state, religion and medicine converged, without forgetting one of the most representative figures of care at the time, that of women as midwives.

For this reason, it was of vital importance to analyse and discuss the primary sources under the dialectic between the official health culture and the popular culture, taking into account social structures; gender as a structural determinant; and religious, economic and symbolic factors and their mentalities [55,56].

The women of this period possessed knowledge of herbs, ointments and concoctions to treat women’s own illnesses, to alleviate the pain of childbirth and to advise on abortive or contraceptive measures as the precursors of the pharmacopoeia [37]. Thus, in the face of their knowledge and their wide range of care, an irrational fear arose on the part of the three great powers of the time: the state, the Church and medicine.

The practices, herbs and remedies known to women were passed down through the female, oral and generational channels [5], which meant that their knowledge escaped the control of the three great powers mentioned above. Their ability to intervene in people’s lives and health by means of magical arts meant that the line separating popular healing practices from witchcraft, superstition and heresy was as thin as that which distinguished a woman from a witch.

Midwives were immersed in the social nucleus in which they assisted [11]. Some were highly trained for the profession, others, even if they were, combined their technical performance with more popular and empirical knowledge typical of the rural world. The latter usually introduced materials, rituals or prayers that were considered superstitious by the Church, although in most cases, the elements were similar to those used in the ecclesiastical sphere [10,37]. This double standard reveals the competition between the powers of the time for social dominance, displacing and condemning the activities carried out by midwives.

One of the reasons for confusion in this period was that anything out of the ordinary was worthy of astonishment and thus of fear, from a child with anatomical differences or having quadruplets, to extreme weather conditions such as excessive cold or flooding [57]. Depending on the degree of intellectual, spiritual and scientific development, these phenomena could be valued within normality. However, given the low literacy and education of the population, they acquired meanings of punishment and curse, signalling that something bad was about to come.

The Royal Pragmatic enacted by Philip II in 1576 prohibited midwives from continuing to be examined and practise freely in the Kingdom of Castile [33]. However, if we take into account the extent of Castile, we can see the significant lack of training and preparation to which women’s health professions were subjected for almost two centuries [35].

Their knowledge and skills enabled them to carry out their three essential functions: teaching, care and religion. They even assisted in normal, complicated and instrumented births, as Inés Martín herself stated [49]. However, these skills were taken away from them with the new regulations of the mid-18th century [33], which gave a boost to the figure of the surgeon-midwife to the detriment of midwives. Many, such as Luisa Rosado [38,39], fought for this situation not to be maintained; nevertheless, this limit of competence, established in the second half of the 18th century, persists to the present day between obstetrics and the speciality of midwifery.

If we look at the social determinants, we see that these women, as well as generally living in rural areas, tended to be single or widowed. They lived in a situation of poverty, which led them to work in various trades in order to survive. They were of advanced age, and even belonged to marginalised social groups, as in the case of Haxa, the Moorish midwife [46,47]. This combination of situations placed women, and in particular those dedicated to caregiving, in a double situation of social and inquisitorial vulnerability.

## 5. Conclusions

The history of nursing and health sciences is a reflection of the history of women, as the female gender has been one of the main backbones of the tradition of care. In the past, the right to access a minimum of literacy and much higher education in different fields of study was reserved for only a privileged section of the population, usually men and sometimes wealthy women.

The profession of midwife was traditionally carried out by women who had achieved an important social value for their knowledge, experience and courage. However, during the period between the 16th and 18th centuries, they found themselves without formal training and as heirs to generational knowledge, techniques and rituals that conflicted with the prevailing culture of that society. Such circumstances turned them into women who transgressed the establishment and were thus judged as sorceresses, superstitious and/or witches.

The general historical and legal context, as well as the background to the period under study (14th–15th centuries), was of great importance, as it provided a glimpse of the drastic social and cultural change. Through this, it can be seen how a greater degree of intellectual, scientific and spiritual development led to the construction of a medieval society that was more tolerant of the exceptional and heterodox. This situation was reversed in the modern era, a period of surrender to the cult of appearance and repression of dissent.

Midwives were the predecessors of nursing and health care today, overcoming difficulties and sociocultural barriers, but they were also condemned for it. They achieved training and an accredited title in the late 15th century, and almost 100 years later, the right to regulated education and trade was taken away from them. Nevertheless, they continued to act as health workers in a society that both demanded them and condemned them for the slightest mistake, without any cover or support, on the contrary, with an inquisitorial network of “witch-hunting”.

The situation of these women reflects the fact that social change cannot only be brought about by professionals and recipients, but requires political transformations that cut across society. History takes us on a journey between before and after, through the legacy received from the anonymous women caregivers in today’s recognised health professions. However, there is still a long way to go in a profession in which the majority are women and in which we still see various phenomena of inequality and inequity within and between professions.

## Figures and Tables

**Table 1:** Thematic blocks related to the references.

Database	Search Strategy	Filter	Points Extracted	References
PubMed Cochrane Cuiden Scopus Web of Science SciELO Books Treatises	history of nursing AND witchcraft midwifery AND witchcraft nursing AND witchcraft history of nursing AND birth setting AND parturition history of nursing AND gender AND legislation Modern Age and nursing midwifery AND inquisition training AND midwifery AND Modern Age midwifery AND Protomedicate witchcraft and Castilla	Last 10 years Article English/Spanish/ French	Training and regulation of the midwifery profession Emergence of new forms of care Midwifery functions Civil and inquisitorial proceedings Amulets at gestation and birth	[19,20,21,22,23,24,25,26,27,28,29,30,31,32] [33,34,35,36,37,38,39] [40,41,42,43,44,45] [46,47,48,49] [50,51,52,53]

Source: authors’ own elaboration.

**Table 2:** Requirements to be met by midwives.

Theoretical–Practical	Moral
Expert in her art Ingenious Able to conduct difficult deliveries	Of good manners Honourable Give good advice and examples Secret Fear God Devoted to the Virgin Mary and other saints and saintesses Good Christian Do not perform spells or superstitions

Source: Authors’ own elaboration based on Carbon, D. [5].

**Table 3:** New regulation of the crafts of partear.

Royal Court of Protomedicine	
Law 10. Compulsory Examination	
Midwife	Surgeon-Midwife
Theoretical and practical examination Eutocic births	Double theoretical–practical examination: midwife and surgeon Complicated and instrumental births

Source: Authors’ own elaboration based on *Book VIII: Sciences, Arts and Crafts*. *Title X* [33].

**Table 4:** List of civil lawsuits against midwives. Chronological order.

Name	Craft	Date	Reason	Whistle-Blower	Accusation	Sentence
Haxa, Muslim	Midwife	1491	Injury	Juana Ruiz (widow)	Sorcery	Sentenced during the trial Acquitted
María García	Midwife	1556	“Hincar un lapo” on the blood of childbirth on the ground	Doctor Tobal	Sorcery	Convicted at trial Public act of faith 200 lashes A fine of 1000 maravedis and disqualification for 6 months Banishment from her jurisdiction
Inés Martín	Midwife	1564	Francisca Galiana and foetus die in childbirth	Mother, husband and daughter of Francisca Galiana	Bad practice	Sentenced during the trial Public act of faith 100 lashes 50,000 maravedis fine Prohibition to exercise her profession Compensation to the family of 10,000 maravedis

Source: Own elaboration from: Archive of the Royal Chancery of Valladolid [26,27].

## Data Availability

Data from this study are available upon request to the research team.

## Abbreviations

DSMC	Dialectical Structural Model of Care
FU	Functional Unit
FF	Functional Framework
EF	Functional Eement
NHA	National Historical Archive
DAC	Diocesan Archive of Cuenca
PARES	Spanish Archives Portal

## References

[1] Browne E.G., Renaud H., La Médecine Arabe (1933). Translator.

[2] El-Madkouri Maataoui M., Zamora Calvo M.J., Ortiz A. (2012). La Representación y Traducibilidad de La Brujería Entre El Árabe y El Español. Espejo de Brujas. Mujeres Transgresoras a Través de la Historia.

[3] Sierra J. (2005). Procesos En La Inquisición de Toledo (1575–1610): Manuscrito de Halle.

[4] Pérez Ramírez D. (1982). Catálogo Del Archivo de La Inquisición de Cuenca.

[5] Carbón D. Libro del arte de las comadres o madrinas, y del regimiento de las preñadas y paridas y de los niños [Internet]. Mallorca: Impreso por Hernando de Cansoles; 1541. http://alfama.sim.ucm.es/dioscorides/consulta_libro.asp?ref=X532352768&idioma=0.

[6] Martín Conty J.L. (2015). Maestros e Instituciones en el Arte de Curar en Toledo Desde el Medivo a la Ilustración.

[7] Sarrión Mora A. (2006). Médicos e Inquisición En El Siglo XVII.

[8] Arce G., Damián J. (2011). Los Proyectos de Ordenanzas Generales de Médicos, Cirujanos y Boticarios de Castilla (ca. 1491–1513). Dynamis.

[9] Beltrán Muñoz C. (2014). El saber obstétrico y ginecológico de las mujeres curanderas y de las matronas en los siglos XV y XVI: Investigación histórica a través de «La Celestina». Matronas Profesión.

[10] Ortiz Gómez T. (1996). Protomedicato y matronas. Una relación al servicio de la cirugía. DYNAMIS Acta Hisp Med Sci Hist Rlw..

[11] García Martínez M.J. (2012). El Oficio de Partera Entre Los Siglos XV al XVIII. Fuentes Documentales Para Su Estudio. Cult. Los Cuid..

[12] Cabré i Pairet M., Ortiz Gómez T. (2001). Sanadoras, Matronas y Médicas En Europa: Siglos XII–XX.

[13] Munn Z., Peters M.D.J., Stern C., Tufanaru C., McArthur A., Aromataris E. (2018). Systematic Review or Scoping Review? Guidance for Authors When Choosing between a Systematic or Scoping Review Approach. BMC Med. Res. Methodol..

[14] Siles J., González C., Martínez F. (2010). Los Cuidados de Enfermería En El Marco de La Historia Social y La Historia Cultural. La Transformación de la Enfermería. Nuevas Miradas Para la Historia.

[15] Siles González J. (2010). Historia Cultural de Enfermería: Reflexión Epistemológica y Metodológica. Av. Enferm..

[16] Siles González J., Solano Ruiz C. (2016). El Modelo Estructural Dialéctico de Los Cuidados. Una Guía Facilitadora de La Organización, Análisis y Explicación de Los Datos En Investigación Cualitativa. CIAIQ2016.

[17] Peters M.D.J., Godfrey C.M., Khalil H., McInerney P., Parker D., Soares C.B. (2015). Guidance for Conducting Systematic Scoping Reviews. JBI Evid. Implement..

[18] Colquhoun H.L., Levac D., O’Brien K.K., Straus S., Tricco A.C., Perrier L., Kastner M., Moher D. (2014). Scoping Reviews: Time for Clarity in Definition, Methods, and Reporting. J. Clin. Epidemiol..

[19] García Martínez M.J., García Martínez A.C.G. (2005). Las Funciones de La Matrona En El Mundo Antiguo y Medieval. Una Mirada Desde La Historia. Matronas Profesión.

[20] Alfonso X., López de Tovar G. (1587). Las Siete Partidas.

[21] De Miguel Sesmero J.R. (2019). La Salud Reproductiva En Las Cántigas de Santa María de Alfonso X El Sabio: Una Visión Desde El Ámbito Médico. Santander Estud. Patrim..

[22] González I. (2009). Posiciones fetales, aborto, cesárea e infanticidio. Un acercamiento a la ginecología y puericultura hispánica a través de tres manuscritos medievales. Miscelánea Mediev. Murc..

[23] Contreras Gil J. (2016). La Formación de Las Matronas: Una Aproximación al Estudio de La Evolución de Esta Profesión, (1857–1957).

[24] Álvarez Nebreda C.C., Álvarez Moya J.M. (2016). Más de Cien Años de Historia.

[25] Córdoba-Flores C. (2020). Instituciones y Políticas de Salud Pública En La Ciudad de México, de La Colonia al Porfiriato. Hist. Rev. Hist. Reg. Local.

[26] Archivo Histórico Provincial de Valladolid (1579). Protocolos, Leg. 583, Fol. 52; Valladolid.

[27] Archivo Histórico Provincial de Valladolid (1573). Protocolos, Leg. 452, Fol. 631; Valladolid.

[28] García Herrero C. (2006). Las Mujeres En Zaragoza En El Siglo XV.

[29] Archivo Municipal de Granada (2005). Libro de Actas Capitulares, No 1, Fol. 61r-61v. La memoria de la Ciudad: El Primer Libro de Actas del Cabildo de Granada (1497–1502).

[30] López de la Plaza G. (1993). Las Mujeres Moriscas Granadinas En El Discurso Político y Religioso de La Castilla Del Siglo XVI (1492–1567). En Esp. Mediev..

[31] Archivo Histórico Nacional (1482). Clero Secular Regular (CSR), Car. 3639, No 5; Valencia.

[32] Carbón D. (1541). Libro Del Arte de Las Comadres o Madrinas, y Del Regimiento de Las Preñadas y Paridas y de Los Niños.

[33] BOE (1805). Libro VIII: De Las Ciencias, Artes y Oficios. Título X: Del Real Protomedicato, y Junta Superior Gubernativa de Medicina. Ley, X. Novísima Recopilación de las Leyes de España, Mandada Formar por el Señor Don Carlos IV.

[34] Gómez-Cantarino S., Romera-Álvarez L., Dios-Aguado M., Ugarte-Gurrutxaga M.I., Siles-González J., Cotto-Andino M. (2022). Queens and Wet Nurses: Indispensable Women in the Dynasty of the Sun King (1540–1580). Healthcare.

[35] Medina A. (1785). Cartilla Nueva Útil y Necesaria Para Instruirse Las Matronas, Que Vulgarmente Llaman Comadres En El Oficio de Partear/Mandada Hacer Por El Real Tribunal Del Protho-Medicato al Doctor Don Antonio Medina.

[36] Ortiz Gómez T. (1996). Protomedicato y Matronas. Una Relación al Servicio de La Cirugía. DYNAMIS Acta Hisp. Med. Sci. Hist. Rlw..

[37] Ganso Pérez A.I. (2017). Las Parteras, Un Arte de Mujeres Para Mujeres.

[38] Archivo Histórico Nacional (1770). Consejos, Leg. 5532, Exp. 25; Madrid, Spain.

[39] Archivo General de Simancas (1765). Gracia y Justicia, Leg. 989, Fol. 687–708; Madrid.

[40] Graham H. (1960). Eternal Eve.

[41] Espina-Jerez B., Domínguez-Isabel P., Gómez-Cantarino S., Pina-Queirós P.J., Bouzas-Mosquera C. (2019). Una excepción en la trayectoria formativa de las mujeres: Al-Ándalus en los siglos VIII-XII. Cult. Los Cuid..

[42] Parker G. (1920). The Early History of Surgery in Great Britain, Its Organization and Development.

[43] García Martínez M.J.G. (2015). Prácticas Ancestrales de Las Matronas Españolas: El “Agua de Socorro” o “Bautismo de Urgencia”. Fuentes Escritas Para Su Estudio. Híades Rev. Hist. Enferm..

[44] Gélis J. (2006). Les Enfants Des Limbes. Mort-Nés et Parents Dans l’Europe Chrétienne.

[45] Foster G.M. (1980). Folklore y Costumbres Del Embarazo, Nacimiento e Infancia. La Antropología Médica En España.

[46] Archivo de la Real Chancillería de Valladolid (1495). Registro de Ejecutorias, Caja 84, 2; Valladolid.

[47] Archivo General de Simancas (1492). Registro General Del Sello (RGS), Leg. 149207, Fol.112; Valladolid.

[48] Archivo de la Real Chancillería de Valladolid (1556). Registro de Ejecutorias, Caja 865, 15.

[49] Archivo de la Real Chancillería de Valladolid (1566). Registro de Ejecutorias, Caja 1091, 7. Real Audiencia y Chancillería de Valladolid.

[50] Forbes T.R. (1962). Midwifery and Witchcraft. J. Hist. Med. Allied Sci..

[51] Archivo Histórico Nacional (1698). Inquisición, Leg. 5323, Exp. 29.

[52] Archivo Histórico Nacional (1648). Inquisición, Leg. 82, Exp. 1.

[53] Forbes T.R. (1966). The Midwife and the Witch.

[54] Real Academia Española (1822). Diccionario de La Lengua Castellana, Reproducción Digital de la 6a Edición.

[55] Siles J. (2010). Historia Cultural de La Enfermería. Av. Enferm..

[56] Espina-Jerez B., Romera-Álvarez L., de Dios-Aguado M., Cunha-Oliveira A., Siles-Gonzalez J., Gómez-Cantarino S. (2022). Wet Nurse or Milk Bank? Evolution in the Model of Human Lactation: New Challenges for the Islamic Population. Int. J. Environ. Res. Public Health.

[57] Córdoba-Flores C. Instituciones y políticas de salud pública en la Ciudad de México, de la Colonia al Porfiriato. HiSTOReLo Revista de Historia Regional y Local [Internet]. 2020;12(24):76-108.

